# Chromosome Y as a marker for sex discrepancies in patients with organ transplants: a case report

**DOI:** 10.1186/s13039-020-00523-0

**Published:** 2021-01-06

**Authors:** Nuria Balaguer, Emilia Mateu-Brull, Roy P. Naja, Jara B. Nagi, Miguel Milán

**Affiliations:** 1 Igenomix Spain Lab S.L.U. Parque tecnológico, Ronda Narciso Monturiol, 11B. Edificios Europark, CP: 46980 Paterna, Valencia Spain; 2 Igenomix UK Ltd, Surrey Technology Centre, Guildford, UK; 3 The Centre for Reproductive & Genetic Health, London, UK

**Keywords:** NIPT, Bone marrow, cfDNA, Sex-discrepancy

## Abstract

**Background:**

Organ transplantations cause discrepancy in results from cell-free DNA (cfDNA) testing, but scientific literature is scarce.

**Case:**

A 33-year old gravida underwent cfDNA testing, which showed high levels of Y chromosome (ChrY) in the maternal bloodstream. The ChrY pattern was comparable to an adult male reference. As a result, cfDNA testing was only informative for autosomes. Routine 20-week ultrasound scan showed no structural alterations and the presence of female external genitalia. Post-clinical research revealed that the patient received a bone marrow transplant from a male donor several years before. Fluorescence in situ hybridization showed that 100% of nuclei analysed from the patient’s lymphocytes presented a ChrY.

**Conclusion:**

This case demonstrates ChrY can be used as a marker to avoid sex discrepancies in certain patients with organ transplants.

## Background

Cell-free DNA (cfDNA) testing for chromosomal aneuploidies offers higher sensitivity and specificity for common autosomal chromosomal aneuploidies and sex determination at an earlier gestational age compared to traditional biochemical and sonographic screening [1]. As a result, cfDNA testing is widely selected as the first choice for detecting common foetal aneuploidies and determining foetal sex [2].

However, it is essential to reinforce the idea that NIPT is still a screening test and that women with a positive screening result require invasive tests to confirm the findings. In addition to the knowledge and understanding the basis of the NIPT and the potential causes of false positive or false negative results, it is essential to enable clinicians and genetic counsellors to counsel the patients comprehensively and appropriately before testing and after receiving the test result. This type of practice ensures the responsible application of NIPT and allows evidence-based decision-making capacity for the future mother.

In this sense, it is critical to transmit the idea that discrepancies between the NIPT and the real genetic dotation of the foetus may arise [3], being these potentially explained by technical human errors and/or biological mechanisms (as previously described) [4]. Amongst sources of human errors include blood sample mislabelling, laboratory methodologic limitations, and suboptimal visualization of the external genitalia associated with limited performance of ultrasound imaging at early gestational ages. In turn, biological reasons for discordance include the presence of a vanishing twin, foetal-placental mosaicism for sex chromosome abnormalities, maternal transplant from a male donor, disorders of sexual development, or other foetal abnormalities associated with anomalous or ambiguous external genitalia [5]. In this context, we describe herein a case where foetal sex determination by noninvasive prenatal testing (NIPT) versus ultrasound screening was discrepant in a pregnant woman who had a bone marrow transplantation from a male donor.

## Case presentation

### Case history

**Fig. 1:**
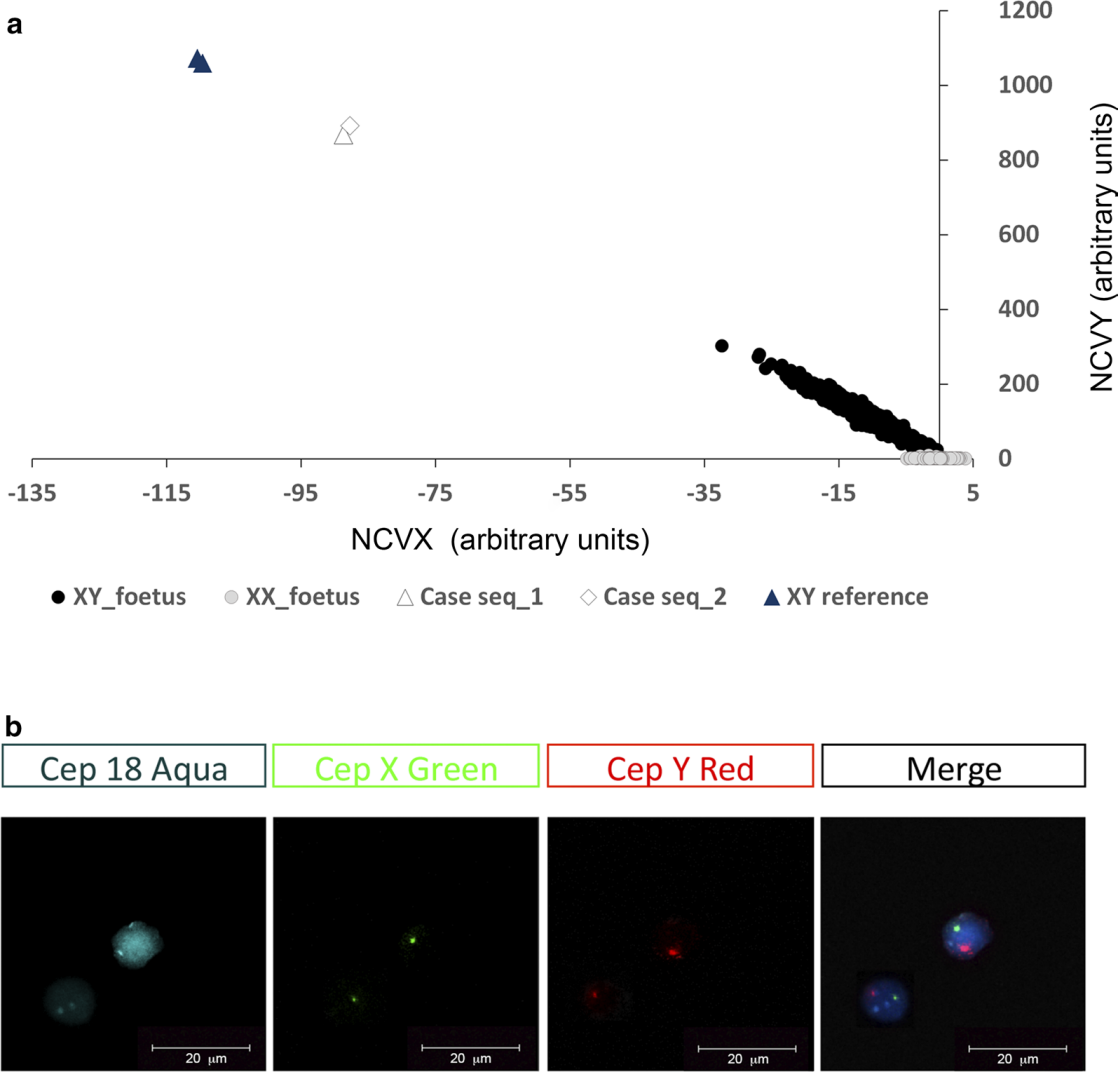
Analysis of Y chromosome presence in maternal circulation. **a** Normalized chromosome value (NCV) for sex chromosomes in singleton XX and XY pregnancies. Male pregnancies exhibit a linear behavior, acquiring higher NCV_Y values as foetal fraction (FF) increases. However, female pregnancies have NCV_Y values closer to 0 and present NCV_X values between − 4 and 4. Note that NCV_Y values detected in maternal circulation of a pregnant woman subjected to a bone marrow transplant are out of scale and are more typical of that expected for an adult male reference sample. **b** Representative micrographs of FISH analysis performed in nuclei isolated from the white blood cell fraction of maternal blood sample. Probes: CEP18-spectrum aqua, CEPX-spectrum green, and CEPY-spectrum red. Positive signals for the CEPY-spectrum red probe confirm presence of the Y chromosome in WBC

The patient was a 33-year-old gravida who conceived after in vitro fertilization treatment in September 2019. At 11 weeks of gestation, she underwent cfDNA testing (NACE test, Igenomix, Valencia, Spain) at her request. The native cfDNA testing algorithm indicated a low risk for trisomies in the analysed chromosomes (13, 18, 21, X, and Y) and a male sex classification. A more detailed analysis of sex chromosomes identified an anomalous normalized chromosome value (NCV) for chromosomes X and Y, compatible with neither a female nor male foetus (NCV_Y = 867.7; NCV_X = − 88.7). Specifically, these values were tenfold higher (in absolute value) than expected for the foetal fraction (FF) registered (6.4%). To exclude a misdiagnosis due to external contamination or possible artifacts, a new specimen was requested for further analysis. Again, the NCV for chromosomes X and Y were concordant with the first cfDNA testing, compatible with a male sex and with a higher than expected NCV_Y value of 891.6 (Fig. 1a). Since FF can be estimated based on amount of Chr Y [6], we compared FF estimations by two different methodologies: the SeqFF approach [7], and another method based on Chr Y [6]. In the first blood sample, SeqFF estimated overall FF of 6.4%, whereas the Y chromosome-based method yielded 73.7%. Similarly, in the second specimen, SeqFF estimation was 8.02%, whereas Y chromosome-based estimation was 75.7%.

Given the data reproducibility, we requested any non-disclosed clinical condition inherent to the patient that could explain our findings. Interestingly, the patient was diagnosed in 2013 with Hodgkin’s lymphoma and underwent 9 months of chemotherapy and radiotherapy. Nevertheless, her tumor relapsed in 2014, and it was decided that stem cell transplantation was her best option. Before proceeding with the treatment, she underwent embryo freezing for fertility preservation and cryopreserved seven embryos. In 2015, she had autologous stem cell transplantation, which was unsuccessful. However, soon after, she successfully received a donor stem cell transplant from her brother. In February 2019, she opted for medicated frozen embryo transfer, where just one embryo was transferred. Preimplantation genetic testing for aneuploidies (PGT-A) was not performed before embryo transfer. Foetal anatomy and nuchal translucency assessment showed no abnormality at 12 weeks of gestation. The foetus showed no structural alterations at the routine 20-week ultrasound scan, although female external genitalia were identified. Following discussion of the consulting physician and patient, further investigations were instigated, including fluorescent in situ hybridization (FISH) analysis of chromosomes 18, X, and Y on the white blood cell (WBC) fraction isolated from a second maternal sample. Of 100 total nuclei analysed, 100% showed two signals for chromosome 18 and one signal each for chromosomes X and Y (Fig. 1b). A female infant was delivered at 37 weeks of gestation by elective Caesarean section and weighed 2.2 kg. There were no reported congenital abnormalities.

## Discussion and conclusions

Foetal sex determination can be carried out reliably from 7 weeks of gestation using real-time quantitative PCR to identify presence or absence of Y chromosome-specific sequences in maternal plasma [8, 9]. Foetal sex also can be reported using cfDNA testing, although it is not considered a primary medical indication. It has been estimated that the overall average sensitivity of using cfDNA to determine foetal sex is 96.6% and overall specificity is 98.9% [10], but these figures may vary depending on the technology and platform used for analysis. Results of cfDNA testing may be discordant with either the ultrasound appearance of external genitalia or PGT-A results for multiple reasons. In this sense, foetal sex discordance rates of 0.0–0.9% have been described [5, 11–13].

While organ transplants are uncommon in women requesting cfDNA testing, transplants have been reported as a source of incorrect foetal sex prediction [3, 14]. To date, case reports detailing the clinical aspects surrounding stem cell or organ transplants are scarce. The largest study published to date was performed by Wardrop et al. [15], who discussed 11 NIPT cases with a previous maternal transplant. Specifically, seven of the cases (63.6%) were bone marrow transplants, but only in one case was a sex discrepancy identified with proper follow-up. From these results, the authors concluded that not all tissues/organs equally contribute cfDNA to maternal plasma, with bone marrow transplants having a more significant contribution to contaminating cfDNA.

Similarly, Balslev-Harder et al. [14] reported that the Y chromosome content in maternal blood was high enough to mask the overall fraction of placental-derived DNA in cfDNA. Interestingly, the authors demonstrated that the length distribution of Y chromosome sequence reads could be used to distinguish rare occurrences of maternal Y chromosomal contribution from leukocytes or possible single organ transplants. In our particular case, we detected that the relative percentages of Y chromosome reads obtained in two independent blood samples presented a distribution similar to an adult male reference. Interestingly, those quantities are very similar to the figures described by others, which state that approximately 75% of cfDNA released to maternal circulation is hematopoietic in origin [16]. Besides, FISH results suggested that all WBCs isolated from the maternal plasma were XY, confirming that the cause of the foetal sex discordance was attributed to the bone marrow transplant.

Similar cases have also been described in other clinical reports where the transplant was from a different organ than bone marrow. Bianchi et al. [17], described their clinical experience with cfDNA testing for foetal sex chromosomes in a large cohort study. Their study identified a discordant result caused by a kidney transplant from a male donor. Also, in the same year, cfDNA testing of a patient who previously received a renal transplant was negative for chromosomal abnormalities and consistent with a male foetus. However, the foetal anatomy ultrasound at 20 weeks of gestation showed female genitalia, confirmed by the birth of a female baby. More recently, a clinical case in 2018 describing cfDNA testing in a woman recipient of a liver transplant from a male donor suggested that graft-derived cfDNA released into the maternal circulation clouded the cfDNA testing prediction of sex [2].

Therefore, given that in certain organ transplants, the amount of sex chromosomes exceeds the expected for the calculated FF, an alert should arise indicating the existence of contaminating DNA in the bloodstream, with the ability to interfere with the final result of the cfDNA testing. Thus, leading companies should modify their algorithms to detect these kinds of scenarios that prevent obtaining informative results for sex chromosomes in terms of aneuploidy calling and correct sex assignment.

Together, these cases demonstrate that due to possible inconsistencies in the final diagnosis, disclosing the foetal sex by cfDNA testing currently might only be advisable when the pregnant mother has had an organ transplant from a female donor. In the remaining scenarios (male donor or donor with an unknown gender), cfDNA testing should be performed only to analyse aneuploidies for autosomes. Therefore, good pre-test counselling and dynamic communication between the health care practitioner and the laboratory performing the cfDNA testing is required for proper management of these cases. In addition, with advancement of new technologies, novel algorithms will be developed that can discern the origin of cell-free DNA, enabling us to exclude interfering graft-derived cell-free DNA from analysis [2, 14].

This case brings to light the importance of accurate cfDNA analysis in cases where a previous organ transplant has occurred, not only due to the possible resultant sex discrepancies but also because of the impact this may have on accuracy of foetal sex aneuploidy detection. In this regard, adequate pre-test counselling to discuss test limitations as well as a good quality ultrasound are mandatory to avoid potential misdiagnoses.

## Data Availability

The data that support the findings of this study are available from Igenomix, S. L., but restrictions apply to the availability of these data, which were used under license for the current study, and so are not publicly available. Data are however available from the authors upon reasonable request and with permission of Igenomix, S.L.

## Abbreviations

cfDNA: Cell-free DNA
ChrY: Chromosome Y
DNA: Deoxyribonucleic acid
FF: Foetal fraction
FISH: Fluorescence in situ hybridization
NCV: Normalized chromosome value
NIPT: Non-invasive prenatal testing
PCR: Polymerase chain reaction
PGTA: Preimplantation genetic testing for aneuploidies
WBC: White blood cell
